# Chemical composition of ethanol extract of *Macrotyloma uniflorum* (Lam.) Verdc. using GC-MS spectroscopy

**DOI:** 10.1186/s13588-014-0013-y

**Published:** 2014-12-02

**Authors:** Sneha Das, Neeru Vasudeva, Sunil Sharma

**Affiliations:** Department of Pharmaceutical Sciences, Guru Jambheshwar University of Science and Technology, Hisar, 125001 Haryana India

**Keywords:** GC-MS, Macrotyloma uniflorum, Chemical constituents, Retention indices

## Abstract

**Background:**

*Macrotyloma uniflorum* Linn (Fabaceae) is a herbaceous plant with annual branches. It is used in kidney stones, inflamed joints, fever, musculoskeletal disorders, sinus wounds and localized abdominal tumors. It is reported as an antioxidant and nutraceutical (forage and food). GC-MS analysis of ethanol extract has led to identification of twenty-eight compounds from *M. uniflorum* by comparison of their retention indices and mass spectra fragmentation patterns with those stored on the GC-MS computer library.

**Results:**

The main constituents identified were mome inositol, ethyl alpha-d-glucopyranoside, n- hexadecanoic acid, linoleic acid (9, 12-octadecadienoic acid), its esters and ethyl derivatives, Vitamin E, stigmasterol and 3-beta-stigmast-5-en-3-ol.

**Conclusions:**

The extracts are rich in linoleic acid and its esters, mome inositol and ethyl alpha-d-glucopyranoside; therefore, this plant can be medicinally beneficial as an antioxidant, in diabetes and its related disorders.

**Electronic supplementary material:**

The online version of this article (doi:10.1186/s13588-014-0013-y) contains supplementary material, which is available to authorized users.

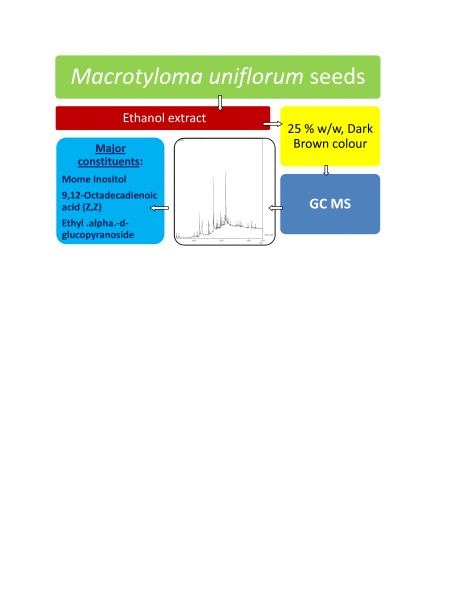

## Background

*Macrotyloma uniflorum* (synonym: *Dolichos biflorus* Linn.) belonging to the family Fabaceae is commonly known as Kulthi in Hindi and horse gram in English. It is a herbaceous plant with annual branches, sub-erect or twining, leaflets of 2.5 to 5 cm. Its seed is 6 to 8 mm long and 3 to 4 mm broad. The seeds are trapezoidal oblong or somewhat rounded in shape and pale to dark reddish brown or orange brown or all black in colour. The genus *Macrotyloma* comprises about 25 species, most of which are restricted to Africa of which four have been identified as *M. uniflorum*, *M. stenocarpum*, *M. verrucosum* and *M. benadirianum*[1]. Traditionally, it has been widely used in the treatment of kidney stones, inflamed joints, fever, musculoskeletal disorders, sinus wounds and localized abdominal tumors [2],[3]. Experimentally, the seeds are reported as hepato-protective, diuretic and antioxidant [4]–[6]. To the best of the authors' knowledge, no published literature exists about the chemical contents of the ethanol extract of *M. uniflorum*. Thus it was planned to carry out GC-MS analysis of ethanol extract of the seeds of *M. uniflorum.*

## Methods

### Preparation of crude extract

The seeds (1 Kg) were coarsely powdered and defatted with petroleum ether (60°C to 80°C) for 7 days by cold maceration. The fat-exhausted drug was further extracted with ethanol (95% *v*/*v*) by soxhlation for 72 h. The extract was concentrated in a rotary vacuum evaporator to yield 25.0% *w*/*w* of dark-brown-coloured extract. The ethanol extract of seeds was diluted with ethanol and filtered with Whatman No. 42 to obtain a particle-free extract for analysis by GC-MS.

### GC-MS analysis

The extract was directly used for the analysis. GC-MS was carried out on a GCMS-QP2010 Plus (Shimadzu, Kyoto, Japan) system with head space sampler (AOC-20s) and auto injector (AOC-20i), equipped with mass selective detector, having ion source temperature of 230°C, interface temperature of 260°C, a solvent cut time of 2.50 min threshold of 1,000 eV and mass range of 40 to 650 m/z. Compounds were separated using a Rtx 5 MS capillary column (Restek Company, Bellefonte, USA: crossbond 5% diphenyl/ 95% dimethyl polysiloxane) having dimensions 30 m (length) × 0.25 mm (diameter) × 0.25 μm (film thickness). The split mode was used at a ratio of 10:1. The temperature of the injector was initialized to 250°C, having a split injection mode. The temperature was programmed from 100°C (3 min), then further increased to 280°C at a ramp rate of 10°C/min (19 min hold).

Helium (>99.999%) was used as the carrier gas at a linear flow velocity of 40.9 cm/s. The debit of gas (helium) vector was fixed to 16.3 mL/min, with a total flow of 1. 21 mL/min. The volume of injected sample was 1.0 μL of ethanol extract. The components were identified by comparison of their retention indices (RI) relative to homologous alkane series (purchased from Sigma, St. Louis, USA) and by comparison of their mass spectral fragmentation patterns with those data provided in WILEY8.LIB, NIST08.LIB, NIST08s.LIB and NIST.LIB. Identification was assumed when a good match of mass spectrum and RI was achieved.

## Results and discussion

The seeds were purchased from Bhagalpur district, Bihar, India, and identified by Dr. K. C. Bhatt, NBPGR, New Delhi. A voucher specimen (PGS-13-02) has been deposited in the Department of Pharmaceutical Sciences (Pharmacognosy Division), Guru Jambheshwar University of Science and Technology, Hisar, Haryana, India.

### Chemical composition

**Figure 1:**
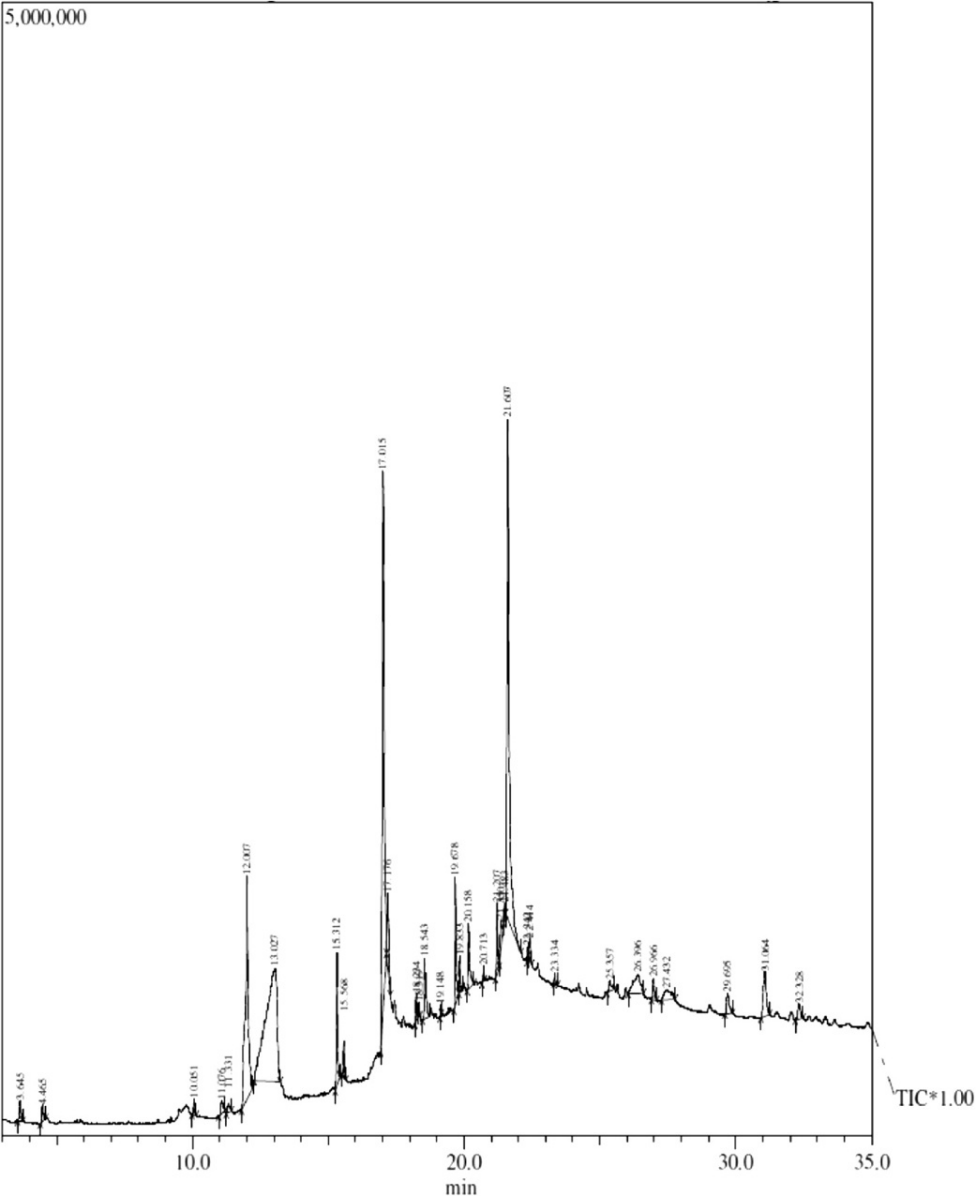
**A typical GC-MS chromatogram of the constituents of ethanol extract of seeds of** ***M. uniflorum*** **.**

GC-MS analysis of ethanol extract led to the identification of twenty-eight compounds from *M. uniflorum* (Table 1, Figure 1)*.* The main constituents identified were two polysaccharides namely mome inositol and ethyl alpha-d-glucopyranoside. A number of fatty acids and their esters have also been identified; they were n- hexadecanoic acid, 9, 12-octadecadienoic acid and its esters. Phytosterols, namely stigmasterol and 3-β-stigmast-5-en-3-ol, were present in traces. Other compounds present were 3-cyclopentylpropionic acid, 2-dimethylaminoethyl ester, heneicosane and vitamin E. literature survey showed that Dolichin A and B and pyroglutaminylglutamine along with some flavonoids were isolated from this plant. Seeds of *M. uniflorum* contain lectins, glycoprotein, agglutinin, an anti-A phytoagglutinin, four glycosidase enzymes, allantoinase and a strong diuretic dipeptide, pyroglutamylglutamine. The seeds are rich source of the enzyme urease and also contain β-sitosterol [7].

**Table 1:** **Chemical composition of ethanol extract of** ***M. uniflorum*** **seed**

Peak	Retention time	Retention indices	Area%	Name
1	3.645	1,056	0.67	Benzeneacetaldehyde
2	4.465	1,116	0.51	Benzeneethanamine
3	10.051	1,499	0.27	L-Phenylalanine, ethyl ester
4	11.076	1,580	0.53	2-(1-methyl-2-propenyl)bicyclo[2.2.1]heptane
5	11.331	1,602	0.39	1H-Pyrrole, 2-(2,4,6-cycloheptatrienyl)
6	12.007	1,660	11.14	Ethyl .alpha.-d-glucopyranoside
7	13.027	1,750	23.24	Mome inositol
8	15.312	1,971	2.76	n-Hexadecanoic acid
9	15.568	1,996	0.49	Heptadecanoic acid, ethyl ester
10	17.015	2,151	19.79	9,12-Octadecadienoic acid (Z,Z)
11	17.176	2,169	1.61	Ethyl (9Z,12Z)-9,12-octadecadienoate
12	18.319	2,300	0.12	Heptadecane, 3-methyl
13	18.543	2,327	1.88	Octanamide, N-(2-hydroxyethyl)
14	19.678	2,465	3.18	3-Cyclopentylpropionic acid, 2-dimethylaminoethyl ester
15	19.833	2,484	0.60	(R)14-Methyl-8-hexadecyn-1-ol
16	20.158	2,526	1.77	Hexadecanoic acid, 2-hydroxy-1-(hydroxymethyl)ethyl ester
17	21.207	2,662	1.27	1-Cyclohexyldimethylsilyloxybutane
18	21.483	2,698	0.22	Eicosane
19	21.607	2,712	19.49	9,12-Octadecadienoic acid (Z,Z)-, 2,3-dihydroxypropyl ester
20	22.342	2,798	0.50	Heneicosane
21	22.414	2,805	0.33	9-Methyl-10,12-hexadecadien-1-ol acetate
22	23.334	2,897	0.19	Hexatriacontane
23	26.396	3,116	2.42	i-Propyl 9,12-octadecenadienoate
24	26.966	3,154	0.72	Vitamin E
25	27.432	3,181	1.76	Tricyclo[20.8.0.0(7,16)]triacontane, 1(22),7(16)-diepoxy
26	29.695	3,290	0.97	Stigmasterol
27	31.064	3,355	2.42	Stigmast-5-en-3-ol, (3 beta)
28	32.328	3,413	0.74	(−)-Isolongifolol, acetate

## Conclusions

Mome inositol, one of the major components of extract of *M. uniflorum*, is reported as anti-alopecic, anti-cirrhotic, anti-neuropathic, cholesterolytic, lipotropic and a sweetener. n-hexadecanoic acids act as a 5-alpha-reductase inhibitor, a hemolytic agent and an antioxidant [8]. (3β)-stigmast-5-en-3-ol has shown an insulin-like effect, that is, stimulating glucose transport apart from its existing cholesterol-lowering efficacy. Therefore, it can play a beneficial role as an antidiabetic agent [9]. Animal studies have revealed that linoleic acid is converted to gamma linoleic acid in the body and can prevent chemically induced diabetes while restoring normal antioxidant status in tissues [10]. It can also prevent diabetic neuropathy, a painful condition resulting from exposure of nerves to high glucose levels [11]. Hence, the plant can be utilized as a natural sweetener, anti-alopecic, anti-cirrhotic, anti-neuropathic, cholesterolytic, lipotropic, antioxidant and antidiabetic.

## Authors’ information

Sneha Das is PhD research scholar pursuing her doctorate under supervision of Professor Neeru Vasudeva and Professor Sunil Sharma at Department of Pharmaceutical Sciences, Guru Jambheshwar University of Science and Technology, Hisar, Haryana, India, 125001.

## Authors’ original submitted files for images

Below are the links to the authors’ original submitted files for images. Authors’ original file for figure 1
