# Supplementary figures and images for: Chemical composition of ethanol extract of Macrotyloma uniflorum (Lam.) Verdc. using GC-MS spectroscopy
Source: Org Med Chem Lett. 2014 Dec 2;4:13. doi: 10.1186/s13588-014-0013-y (PMC4970433; doi:10.1186/s13588-014-0013-y)

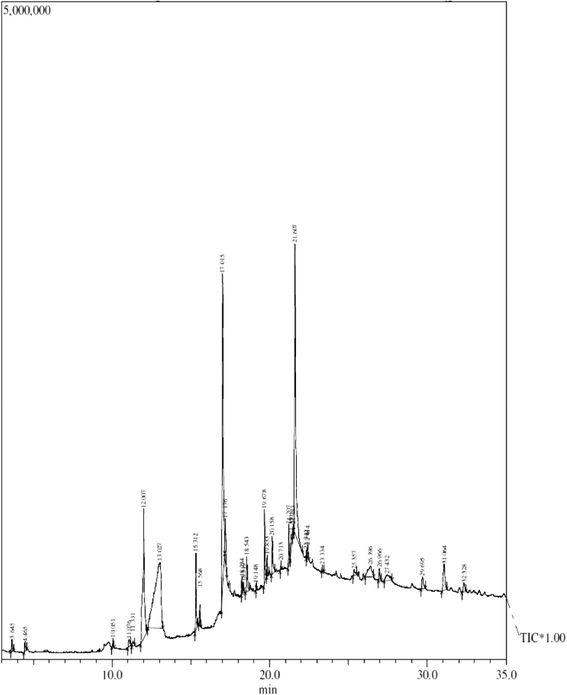

Supplement: Supplementary file 1 — Authors’ original file for figure 1 [file 13588_2014_13_MOESM1_ESM.gif]
